# Hedgehog Signaling Controls Chondrogenesis and Ectopic Bone Formation via the Yap-Ihh Axis

**DOI:** 10.3390/biom14030347

**Published:** 2024-03-14

**Authors:** Qian Cong, Yingzi Yang

**Affiliations:** Department of Developmental Biology, Harvard School of Dental Medicine, Harvard Stem Cell Institute, Boston, MA 02115, USA; yingzi_yang@hsdm.harvard.edu

**Keywords:** heterotopic bone formation, Hedgehog signaling, Ihh, osteoblast, human diseases, FOP

## Abstract

Fibrodysplasia ossificans progressiva (FOP) is a rare congenital disorder characterized by abnormal bone formation due to ACVR1 gene mutations. The identification of the molecular mechanisms underlying the ectopic bone formation and expansion in FOP is critical for the effective treatment or prevention of HO. Here we find that Hh signaling activation is required for the aberrant ectopic bone formation in FOP. We show that the expression of *Indian hedgehog* (*Ihh*), a Hh ligand, as well as downstream Hh signaling, was increased in ectopic bone lesions in *Acvr1^R206H^*; *ScxCre* mice. Pharmacological treatment with an Ihh-neutralizing monoclonal antibody dramatically reduced chondrogenesis and ectopic bone formation. Moreover, we find that the activation of Yap in the FOP mouse model and the genetic deletion of *Yap* halted ectopic bone formation and decreased *Ihh* expression. Our mechanistic studies showed that Yap and Smad1 directly bind to the Ihh promoter and coordinate to induce chondrogenesis by promoting *Ihh* expression. Therefore, the Yap activation in FOP lesions promoted ectopic bone formation and expansion in both cell-autonomous and non-cell-autonomous manners. These results uncovered the crucial role of the Yap-Ihh axis in FOP pathogenesis, suggesting the inhibition of Ihh or Yap as a potential therapeutic strategy to prevent and reduce HO.

## 1. Introduction

Heterotopic ossification (HO), characterized by the abnormal formation of bone outside of the skeletal structure, often occurs as a complication following injury or as a manifestation of specific genetic disorders [1]. It causes a progressive loss of mobility, locking joints with debilitating physical pain, and leads to a reduced lifespan. This abnormal bone growth may occur in multiple soft tissues, leading to stiffness in the affected areas and limiting movement [1,2].

Fibrodysplasia ossificans progressiva (FOP) [3] and progressive osseous heteroplasia (POH) [4] are two forms of genetic HO, which are characterized by the extensive and progressive replacement of soft tissues with bone. FOP is caused by a highly conserved activating mutation (R206H) in the glycine–serine (GS)-rich regulatory domain of the bone morphogenetic protein (BMP) type I receptor, Activin-like kinase 2 (ALK2 or ACVR1) [5,6,7,8]. It has been shown that the FOP mutant receptor, ACVR1 (R206H), displays heightened sensitivity to BMP ligands [9,10] and activins [11,12]. Currently, most drug candidates in clinical trials for FOP treatments, such as Saracatinib, DS-6016a, and BLU-782, suppress the abnormal activation of BMP signaling [13]. However, as physiological BMP signaling is critical for homeostasis, indiscriminately blocking BMP signaling to treat FOP may cause some problems. Sohonos (palovarotene) [14], the retinoic acid receptor agonist, has been approved by the U.S. Food and Drug Administration (FDA) for reducing the volume of new ectopic bone formation in adults and children with FOP. This approval represents a significant milestone in FOP treatment. New mechanistic findings in the understanding of FOP pathogenesis may offer novel therapeutic strategies and opportunities for intervention. These could potentially lead to the development of more targeted and effective treatments for FOP and its related disorders characterized by abnormal bone formation in soft tissues.

Hh signaling is a pivotal pathway in chondrogenesis, the process of cartilage formation during embryonic development [15]. Investigations into neural crest cell dynamics and Hh signaling have elucidated its crucial role in craniofacial development and skeletal formation [16]. The mutation in the Ihh variant alters Gli1–DNA binding patterns and weakens cellular proliferation and migration processes, thereby deepening our understanding of the pathogenesis of Brachydactyly type A1 (BDA1) and the involvement of Ihh signaling in cartilage development [17]. Additionally, it is reported that Ihh signaling plays a critical role in mediating hypertrophy in both mesenchymal stem cells (MSCs) and human articular chondrocytes [18]. Notably, a Hedgehog agonist treatment has showed promise in enhancing tendon-to-bone healing after rotator cuff repair by stimulating mineralized fibrocartilage production [19]. Ihh is required for endochondral ossification [20], which is found in FOP [6]. The Hh signaling pathway has been found to interact with the Bmp signaling pathway. For instance, there is direct association of Smad1 with truncated Gli3 protein [21], underscoring the involvement of Smad proteins in the crosstalk between these signaling pathways. In vivo findings from *Smad1/5* conditional knockout mice indicate that the BMP regulation of *Ihh* is direct and Smad1/5-dependent, as evidenced by their significantly reduced *Ihh* expression [22]. Additionally, multiple studies suggest that *Ihh* is a direct target gene of BMP signaling [23] and that Ihh signaling itself also promotes *BMP* expression. Smad4 can directly bind to the GC-rich motifs of the *Ihh* promoter, suggesting that BMP signaling via Smads stimulates *Ihh* expression [23].

In this study, we set up a conditional knock-in model of FOP, in which the expression of *Acvr1^R206H^* from the endogenous *Acvr1* locus is dependent on Cre-mediated recombination. Using this genetic model of FOP, we found that both Hh signaling and Yap were activated in chondrogenic lesions and the ectopic bone region. A pharmacological treatment with an Ihh-neutralizing monoclonal antibody (mAb) dramatically reduced ectopic bone formation without affecting normal bone mass. The genetic deletion of *Yap* halted HO and decreased *Ihh* expression. Mechanistic studies identified two Smad1 binding sites on the *Ihh* promotor and one Yap/Tead4 binding site, which is close to one of the identified Smad1 binding sites. Moreover, Yap and Smad1 can bind to each other and coordinate to induce chondrogenesis by promoting *Ihh* expression. Together, these data show that Ihh is required for the endochondral ossification in FOP and that Yap activation, via *Ihh* expression, promotes ectopic bone’s formation and expansion.

## 2. Materials and Methods

### 2.1. Mice

All mouse experiments were approved by the Institutional Animal Care and Use Committee at Harvard Medical School. Both male and female mice were included in our studies, and we did not see a gender difference in HO. All the mice used in these experiments were housed four per cage, fed a diet of normal chow and water, and exposed to light for 12 h daily. All mice are described in published research: *Yap^f/f^* [24], *ScxCre* [25], and *Acvr1^R206H/+^*(*Acvr1^FlExR206H/+^*) [25]. Dr. Aris Economides from Regeneron Pharmaceuticals, Inc., kindly supplied the *Acvr1^FlExR206H^* mice, which conditionally express *Acvr1^R206H^* knock-in alleles [25]. *Acvr1^FlExR206H/+^*; *ScxCre* mice were treated with Ihh mAb (Abnova, Taiwan, M01, 200 ng/mL/d intraperitoneal (IP) injection, 5 days per week) or an equal volume of PBS for 8 weeks, starting at 4 weeks of age.

### 2.2. Tenocytes’ Isolation and Culturing

The patellar tendon and Achilles tendon were cut out of one-month-old wildtype and *Acvr1^R206H^* mice. Muscle and other tissues were carefully cleaned from the tendon and it was washed with cold PBS several times before being cut into small pieces. All the tendons were transferred into a 2 mL tube containing 2 mL of collagenase type I (3 mg/mL) and dispase (4 mg/mL) and it was placed on a rotator installed in an incubator set at 37 °C. The cells were collected twice every 30 min, until all the tissue was digested.

### 2.3. Micro-CT Scanning

The tibia and femur were collected and fixed overnight in 4% paraformaldehyde (PFA) and subsequently preserved in 70% ethanol before undergoing high-resolution micro-CT analysis using a desktop μCT35 (Scanco Medical, Brüttisellen, Switzerland). The scanner parameters were set at 55 kV, 145 μA, and 8W, utilizing an 11.5 mm × 75 mm holder. Analysis of both ectopic and endogenous bone’s microarchitecture was conducted, including parameters such as bone volume fraction (BV/TV), trabecular number (Tb.N), trabecular thickness (Tb.Th), and trabecular separation (Tb.Sp). Reconstruction of 3D images and data analysis were performed using μCT V6.1 software. Normalization was achieved using a standard phantom for consistency in measurements.

### 2.4. Von Kossa and Safranin O Staining

Sections were washed in distilled water and then stained with 5% silver nitrate solution under a 60-watt lamp for 1 h. After staining, sections were washed three times in distilled water, followed by a 5 min staining in 5% sodium thiosulfate solution and a final rinse in water to remove excess staining solution and any residual reagents. For safranin O and fast green staining, sections were washed three times. Then, they were stained with 0.2% fast green for 5 min, followed by 0.1% safranin O staining for 20 min.

### 2.5. Adenovirus Treatment

Cre recombinase adenovirus (Baylor College of Medicine, Houston, TX, USA, ∼10^12^ pfu/mL) was diluted 1:2000 to infect cells. Fresh medium was added four hours post-infection. Then, the medium was changed after 24 h.

### 2.6. qRT-PCR

Total RNA extraction was carried out using the TRIZOL reagent (Life Technologies, Carlsbad, CA, USA) following the manufacturer’s instructions. Subsequently, first-strand cDNA synthesis was performed using SuperScript II Reverse Transcriptase with random primers (Life Technologies) on 1–3 μg of total RNA. Quantitative real-time PCR (qRT-PCR) was conducted using an SYBR Select Master Mix on a StepOnePlus thermal cycler from Applied Biosystems. Expression levels were normalized to glyceraldehyde 3-phosphate dehydrogenase (GAPDH). Data analysis was based on three biological replicates, with technical replicates performed for each sample to ensure experimental consistency. The primer sequences are shown in Table S1.

### 2.7. Immunofluorescent Staining

Tissues were fixed in 4% paraformaldehyde in phosphate-buffered saline (PBS) and subsequently processed for high-quality cryosections of undecalcified adult rodent bones [26]. The sections were then blocked using 10% donkey serum and 0.5% Triton X-100 in PBS before proceeding with immunohistochemistry. Primary antibodies were applied, including anti-Yap (Cell Signaling Technology, Danvers, MA, USA, Cat# 14074S), anti-Shh (Developmental Studies Hybridoma Bank, lowa, CA, USA, Cat# 5E1), anti-Opn (R and D systems, Minneapolis, USA, Cat# AF808), Anti-SP7 (Abcam, Cambridge, UK, Cat# ab22552), anti-Ihh (Santa Cruz, Santa Cruz, CA, USA, sc-271101), and anti-Sox9 (EMD Millipore, Burlington, VT, USA, AB-5535), followed by their corresponding secondary antibodies. Finally, the sections were mounted using a mounting medium containing DAPI (Sigma-Aldrich, Burlington, VT, USA, F6057) to visualize cell nuclei.

### 2.8. ChIP-PCR

The control and *Acvr1^R206H^* tenocytes were infected with Ad-Cre. ChIP assays were conducted using a ChIP-seq enzymatic kit (Chromatrap, Norfolk, UK, Product #500192). Chromatin was immunoprecipitated using an anti-Tead4 antibody (Santa Cruz, Santa Cruz, CA, USA, sc-101184) and an anti-Smad1 antibody (Cell Signaling Technology, Danvers, MA, USA, Cat#14074S). The ChIP-derived DNA was quantified using a quantitative real-time PCR (qRT-PCR). The primer sequences are shown in Table S1.

### 2.9. Co-Immunoprecipitation (Co-IP)

The control and *Acvr1^R206H^* tenocytes were lysed and proteins were collected and cleaned. Then, the lysate was incubated with IgG (BioLegend, San Diego, CA, USA, Cat#401501), Yap (Santa Cruz, Santa Cruz, CA, USA, sc-101199), and Smad1 (Cell Signaling Technology, Danvers, MA, USA, Cat#14074S) primary antibodies, respectively, at 4 °C overnight, allowing for specific antibody–protein binding. Immunoprecipitation was then carried out by adding Dynabeads Protein G (Invitrogen, Waltham, MA, USA, Cat#10004D) for 2 h at 4 °C. Subsequent washes removed unbound proteins, and the complexes were eluted from the beads for further analysis.

### 2.10. Statistical Analysis

Quantifications were conducted using at least three independent experimental groups. Statistical analyses comparing groups involved a two-tailed Student’s t-test when only two groups were compared, while a one-way ANOVA with Tukey’s post hoc test were employed for comparisons between multiple groups. A significance level of *p* < 0.05 was considered statistically significant. Error bars on all graphs represent the standard deviation (SD) of the mean unless otherwise specified.

## 3. Results

### 3.1. Hedgehog Signaling Is Activated in FOP Mouse Models

**Figure 1:**
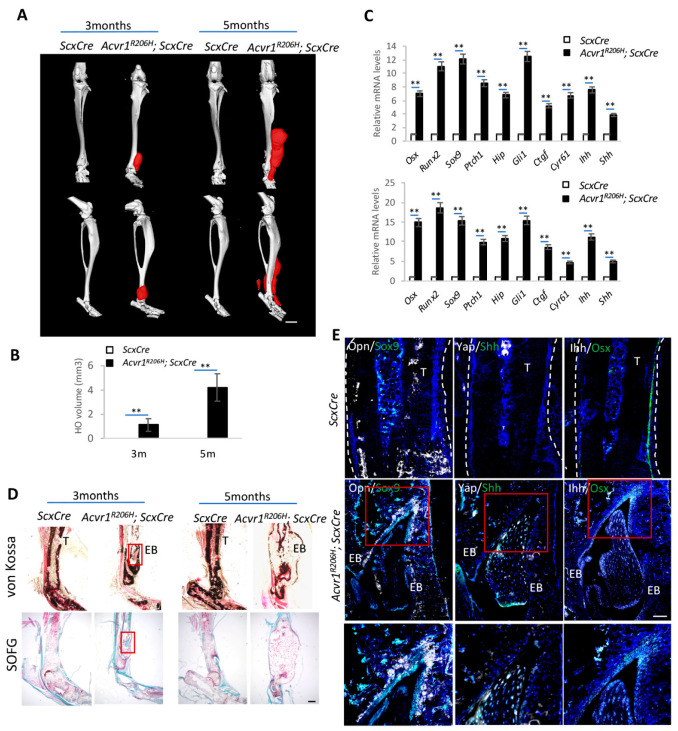
Activation of Hh signaling is detected in the FOP mouse model. (**A**) Representative micro-CT images of *Acvr1^R206H^; ScxCre* and *ScxCre* mice at indicated time points. Ectopic bone is indicated in red. N = 6 biological replicates. Scale bar: 1 mm. (**B**) Quantification of ectopic bone volume from (**A**) ** *p* < 0.01, one-way ANOVA followed by Tukey’s multiple comparisons tests. (**C**) Gene expression analysis by qRT-PCR (mean ± SD; N = 3 biological replicates). (**D**) Representative von Kossa and SOFG staining of tissue sections. Scale bar: 100 μm. (**E**) Representative immunofluorescent images of Opn, Sox9, Yap, Shh, Ihh, and Osx in the ectopic bone sections of the indicated mice. Lower panel: higher-magnification images of the boxed regions. DAPI stained the nuclei. N = 3 biological replicates. Scale bar: 100 μm.

Our previous studies demonstrated that Hedgehog signaling is both necessary and sufficient to induce ectopic bone formation in POH mouse models [27]. Additionally, the upregulated expression of Hh ligands is found not only in the genetic HO mouse models of POH and FOP, but also in injury-induced mouse models [28]. In order to test whether Hh signaling plays as a central role in FOP, we set up a FOP mouse model by generating *Acvr1^R206H^*; *ScxCre* mice, in which a BMP type I receptor is conditionally activated in tendon/ligament cells. Three-month-old and five-month-old control and *Acvr1^R206H^*; *ScxCre* mice were collected and analyzed by micro-CT scanning to identify their ectopic bone formation (Figure 1A). Their volume of HO was much increased from 3 months to 5 months based on a quantification of their HO volume (Figure 1B), indicating progressive HO expansion, which is consistent with other studies, including our own [25,28]. The expression of the osteogenic markers *Osx*, *Runx2*, and *Sox9*; the Hh signaling target genes *Gli1*, *Hhip*, and *Ptch1*; the Yap target genes *Ctgf* and *Cyr61*; and the Hh ligands *Ihh* and *Shh* were much increased in HO lesions (Figure 1C). In the FOP mouse model, the expression of Ihh is higher than Shh, consistent with the important role of Ihh in endochondral ossification.

As ectopic bone formation in FOP is governed by endochondral ossification, safranin O/fast green (SOFG) and von Kossa staining were performed to evaluate chondrogenesis and ectopic bone formation, respectively (Figure 1D). In addition, the immunofluorescence (IF) staining of Sox9, Opn and Osx was performed (Figure 1E). Ihh, Shh, and Yap expressions were also determined by IF staining to assess the cells’ Hedgehog and Yap activities (Figure 1E). Ihh and Shh were highly expressed in the Osx^+^, Sox9^+^ and Yap^+^ ectopic chondrocytes and ectopic osteoblast cells. Our recently published data reveal that HO expansion is a non-cell-autonomous process driven by the Yap-Shh positive feedback loop [28]. The co-staining of Shh and Yap shows consistent results with our previous findings; however, the expression of Ihh is much higher in FOP models, indicating Ihh may play a more important role in driving chondrogenesis to promote endochondral bone formation in this case.

### 3.2. Ihh Is Required during Chondrogenesis in FOP Mouse Models

**Figure 2:**
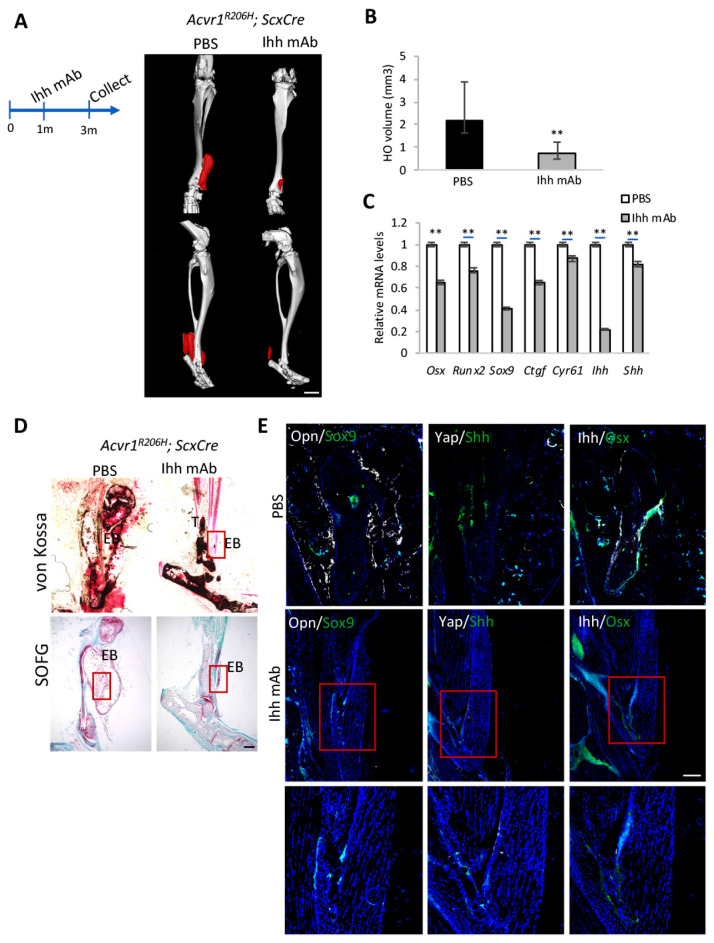
Ihh plays an essential role in chondrogenesis in FOP mouse models. (**A**) Representative micro-CT images of *Acvr1^R206H^; ScxCre* mice with PBS or Ihh mAb (Abnova; M01) treatment at indicated time points. Ectopic bone is indicated in red. N = 5 biological replicates. Scale bar: 1 mm. (**B**) Quantification of ectopic bone volume from (A) ** *p* < 0.01, one-way ANOVA followed by Tukey’s multiple comparisons test. (**C**) Gene expression analysis by qRT-PCR (mean ± SD; N = 3 biological replicates). (**D**) Representative von Kossa and SOFG staining of tissue sections. Scale bar: 100 μm. (**E**) Representative immunofluorescent images of Opn, Sox9, Yap, Shh, Ihh, and Osx in the ectopic bone sections of the indicated mice. Lower panel: higher-magnification images of the boxed regions. DAPI stained the nuclei. N = 3 biological replicates. Scale bar: 100 μm.

To determine whether increased Ihh is required for HO and its expansion, Ihh mAb [29] was applied, via an intraperitoneal (IP) injection, to 1-month-old *Acvr1^R206H^*; *ScxCre* mice when no ectopic bone was detected. The tissue was collected from 3-month-old mice, followed by micro-CT scanning and qRT-PCR analysis (Figure 2A–C). Measurements of their ectopic bone volume based on micro-CT images confirmed that ectopic bone was dramatically reduced by the Ihh mAb treatment. The expression of *Sox9*, *Osx*, *Runx2*, and the Yap target genes *Ctgf* and *Cyr61* (Figure 2C) was also reduced, indicating that chondrocyte and osteoblast differentiation are halted by the Ihh mAb treatment. It is interesting to find that Yap activities were also reduced by Ihh mAb. Previous studies by our lab showed the Yap-Shh feedback loop promoting ectopic bone expansion in both genetic and injury-induced HO [28], based on qRT-PCR data. Shh levels were also reduced due to the Yap target gene decreases after the Ihh mAb treatment (Figure 2C). Von Kossa and SOFG staining showed much-reduced ectopic bone lesions and chondrogenesis after the Ihh mAb treatment (Figure 2D). Fluorescence images revealed the colocalization of Ihh^+^/Osx^+^ and Yap^+^/Shh^+^ during the chondrogenic process and ectopic bone formation. After two months of the Ihh mAb treatment, cells expressing Opn, Sox9, and Yap were much decreased (Figure 2E).

To test whether the Ihh mAb treatment could reduce endogenous bone mass and therefore cause osteoporosis, we quantified the endogenous bone mass, trabecular bone number, and trabecular bone thickness of the mice, and found that they were not reduced after two months of continuous Ihh mAb administration (Figure S1), suggesting that the bone reduction effects of Ihh mAb are largely confined to HO. To assess its impact on longitudinal growth, we measured the bone length of *Acvr1^R206H^*; *ScxCre* mice following their treatment with an Ihh mAb, as bone length is primarily regulated by the Ihh-controlled growth plate [20]. The quantification data showed the bone length was slightly decreased compared to the control mice (Figure S2). Taken together, these data showed that Ihh is required for chondrogenesis and the ectopic bone formation process in FOP. Our results also identify Ihh inhibition as a potential therapeutic strategy for HO reduction, with limited influence on endogenous bone.

### 3.3. Yap Activation Promotes Ihh Expression

**Figure 3:**
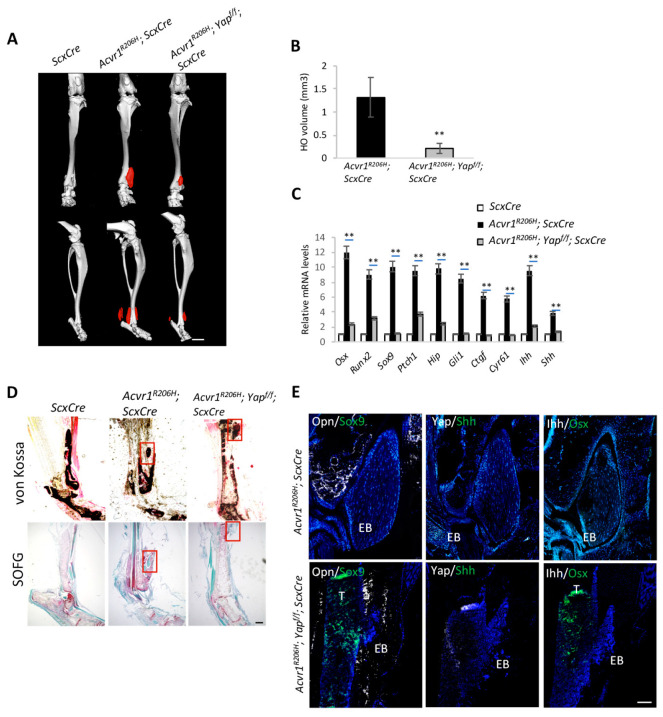
Yap is required for *Ihh* expression and ectopic bone formation. (**A**) Representative micro-CT images of *Acvr1^R206H^; ScxCre*, *Acvr1^R206H^; Yap^f/f^; ScxCre,* and *ScxCre* mice. Ectopic bone is indicated in red. N = 4 biological replicates. Scale bar: 1 mm. (**B**) Quantification of ectopic bone volume from (**A**) ** *p* < 0.01, one-way ANOVA followed by Tukey’s multiple comparisons test. (**C**) Gene expression analysis by qRT-PCR (mean ± SD; N = 3 biological replicates). (**D**) Representative von Kossa and SOFG staining of tissue sections. Scale bar: 100 μm. (**E**) Representative immunofluorescent images of Opn, Sox9, Yap, Shh, Ihh, and Osx in the ectopic bone sections of the indicated mice. N = 3 biological replicates. Scale bar: 100 μm.

In light of our previous studies revealing that a loss of *Gnas* leads to an activation of Yap transcription activity, which directly drives *Shh* expression, the pharmacological inhibition of Yap with verteporfin (VP) [30] abolished HO in genetic and injury-induced HO models [28]. To test whether Yap is also required for HO and its expansion in the FOP mouse model, we set up the *Acvr1^R206H^*; *Yap^f/f^*; *ScxCre* mice to genetically delete *Yap*. The legs of 3-month-old mice were collected and subjected to micro-CT scanning and qRT-PCR analysis (Figure 3A,C). The measurements of ectopic bone volume based on micro-CT images and von Kossa staining (Figure 3B,D) showed much decreased HO, as well as a decreased expression of *Osx*, *Runx2*, *Sox9,* and *Ihh* (Figure 3C), suggesting that Yap regulates chondrocyte and osteoblast differentiation via promoting *Ihh* expression. Consistently, Opn^+^ and Sox9^+^ cells were decreased compared to the control (Figure 3E). Safranin O staining also showed that *Yap* loss reduced ectopic chondrocyte differentiation (Figure 3D). Fewer Yap^+^ and Ihh^+^ cells were detected around the ectopic lesions (Figure 3E). Taken together, these data demonstrate that Yap activation is required to induce *Ihh* expression and promote ectopic cartilage and bone formation in FOP.

### 3.4. Ihh and Yap Are Activated in Acvr1^R206H^ Tenocytes

**Figure 4:**
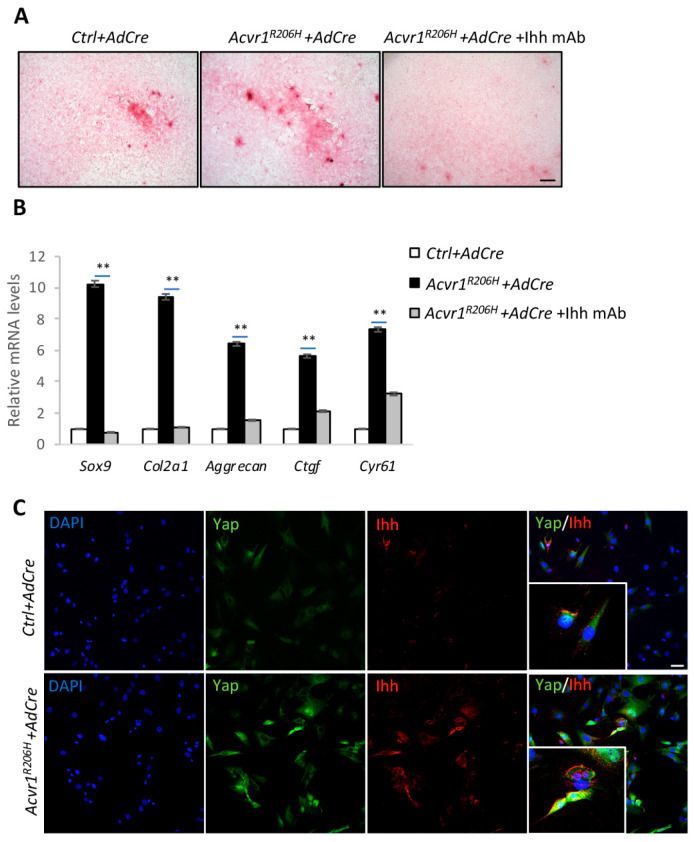
Ihh mAb inhibits chondrogenesis and Yap activities in the *Acvr1^R206H^* tenocytes. (**A**) Safranin O staining of *Ctrl* and *Acvr1^R206H^* tenocytes, with Ad-Cre infection and treatment with Ihh mAb. N = 3 biological replicates. Scale bar: 100 μm. (**B**) qRT-PCR analysis of indicated tenocytes treated with chondrocyte-inducible medium. ** *p* < 0.01, one-way ANOVA followed by Tukey’s multiple comparisons test. (**C**) Representative immunofluorescent images of Yap and Ihh from *Ctrl* and *Acvr1^R206H^* tenocytes cultured in chondrocyte-inducible medium. Different channels (DAPI, Yap, and Ihh) and merged images are shown. Higher magnification is shown in the left lower panel. N = 3 biological replicates. Scale bar: 20 μm.

To test Yap and Ihh expression in vitro, tenocytes were isolated from 1-month-old *Acvr1^FlExR206^* mice. The tenocytes were cultured for seven days before Adenovirus Cre (AdCre) infection to induce the expression of *Acvr1^R206H^*. Safranin O staining showed much stronger chondrocyte differentiation in the *Acvr1^R206H^* group compared to the control (Figure 4A). The expression of chondrocytes’ markers, *Sox9*, *Col2a1*, and *Aggrecan,* were much increased. The expression of *Ihh* and the Yap target genes *Ctgf* and *Cyr61* were also increased (Figure 4B). Additionally, the Ihh mAb treatment blocked chondrogenesis in *Acvr1^R206H^* tenocytes, as well as the chondrocytes’ markers (Figure 4A,B). Interestingly, Ihh mAb also decreased the Yap target genes’ expression, suggesting that Ihh could also regulate the Yap activities in tenocytes (Figure 4B). Fluorescence images of the cultured tenocytes showed the nuclear localization of Yap and increased Ihh, indicating the activation of Yap in *Acvr1^R206H^* tenocytes (Figure 4C). These data confirmed the upregulation and colocalization of Yap and Ihh, suggesting the direct regulation of Yap and Ihh in FOP models.

### 3.5. Yap Directly Activates Ihh Expression to Promote HO

**Figure 5:**
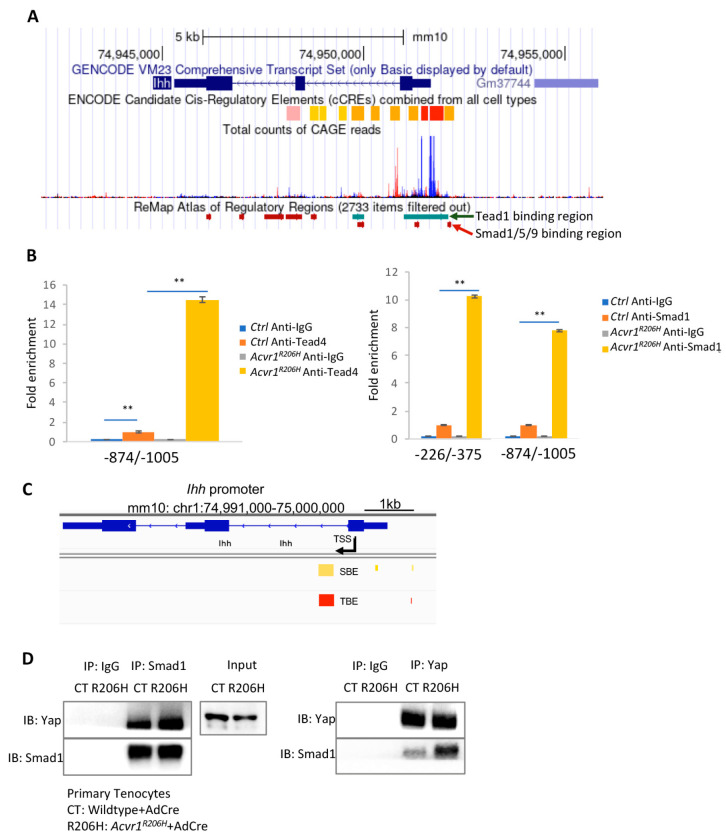
Ihh is a direct target of the Yap transcription factor. (**A**) The candidate cCREs and the ReMap atlas of regulator regions analyzed from the UCSC genome browser. (**B**) ChIP-qPCR analysis of the Yap/Tead4 and Smad1 binding sites on the *Ihh* promoter. (mean ± SD; N = 3 biological replicates.) ** *p* < 0.01, one-way ANOVA followed by Tukey’s multiple comparisons test. (**C**) TBE and SBE analysis of the *Ihh* promoter. Red, TBE; yellow, SBE. (**D**) Co-IP analysis in control and *Acvr1^R206H^* tenocytes.

The ectopic *Ihh* expression regulated by Yap guided us to look for genome-wide gene-expression changes. The ENCODE candidate cis-regulatory elements (cCREs) of Ihh were identified by the UCSC genome browser, including their promoter, distal, and proximal enhancers (Figure 5A). The ReMap atlas of regulatory regions from the Chromatin Immunoprecipitation-quantitative (ChIP) sequencing data of all cell types was shown, including several Yap/Tead binding elements (TBEs) and Smad1/5/9 binding elements (SBEs). Specifically, in the promoter region, TBEs in the fibroblasts and SBEs in the hair follicles were identified (Figure 5A), indicating that both Yap and Smad1/5/9 are involved in the transcriptional regulation of Ihh.

To gain mechanistic insight into the molecular relationship between Yap activation and *Ihh* expression in chondrogenesis, we isolated tenocytes from control and *Acvr1^R206H^* mice. We cultured the wildtype and *Acvr1^R206H^* tenocytes and subsequently infected them with AdCre. To test whether Ihh is a direct transcriptional target of Yap, ChIP-qPCR was performed in the control and *Acvr1^R206H^* tenocytes (Figure 5B). The promoter region of Ihh was tested using multiple primers. We identified a Yap/Tead4 binding site (−874/−1005) on the Ihh promoter, which was included in the previously identified Tead binding region in fibroblasts (Figure 5A), demonstrating that Ihh is a direct transcriptional target of Yap.

Multiple studies by other groups have shown that Ihh is a direct target gene of BMP, dependent on Smad1/5 [31]. Smads can directly bind to the GC-rich motifs of the Ihh promoter (0/−423), suggesting that BMP stimulates *Ihh* expression [23]. To test whether Ihh is a direct transcriptional target of Smad1, a ChIP-qPCR was performed in control and *Acvr1^R206H^* tenocytes (Figure 5B). Two binding sites of Smad1 (−226/−375 and −874/−1005) were identified on the Ihh promoter, demonstrating that Ihh is a direct transcriptional target of Smad1.

To further evaluate the possibility that Yap/Tead4 coordinates with Smad1/5/9 on the Ihh promoter, we remapped the identified SBEs and TBEs using ChIP-PCR, which were identified by us in *Acvr1^R206H^* tenocytes, where we found that one of the SBEs on the Ihh promoter is very close to the TBEs (Figure 5C). To confirm the direct protein binding of Yap and Smad1, co-immunoprecipitation (co-IP) was performed in control and *Acvr1^R206H^* tenocytes. It shows that Yap and Smad1 can bind to each other, coordinating to induce *Ihh* expression and chondrogenesis (Figure 5D). Taken together, these data demonstrate that Yap and Smad1 can directly activate *Ihh* expression by binding to the Ihh promoter, coordinating to promote chondrogenesis and ectopic bone formation and expansion in FOP mouse models.

## 4. Discussion

This study delves into the intricate molecular mechanisms governing ectopic bone formation in FOP, a condition characterized by heterotopic ossification due to gain-of-function mutations in the BMP type I receptor ACVR1. The research focuses on the role of Hh signaling, and particularly the Hh ligand Ihh, in driving chondrogenesis and ectopic bone formation in FOP, shedding light on potential therapeutic interventions.

Firstly, Hh signaling is activated in FOP mouse models with a notable increase in *Ihh* expression in their ectopic bone lesions. Unlike in POH mouse models, Ihh is found to play a more significant role than Shh in FOP, emphasizing its importance in endochondral ossification. To validate the critical role of Ihh, Ihh mAb was administrated to FOP mouse models, demonstrating a substantial reduction in chondrogenesis and ectopic bone formation. This pharmacological intervention proves the central role of Ihh in these processes without affecting endogenous bone mass, indicating that Ihh mAb could serve as a potential therapeutic strategy for reducing heterotopic ossification in FOP patients. In order to minimize the side effects caused by Ihh mAb’s administration, different dosages and treatment strategies would be tested in future work.

Previous studies have shown that Ihh signaling is a critical molecular pathway that regulates various aspects of chondrogenesis, including the proliferation and differentiation of chondroprogenitor cells. The dysregulation of Ihh signaling can lead to skeletal abnormalities and is associated with disorders such as chondrodysplasias [20]. Also, it has been reported that an Ihh/parathyroid hormone-related peptide (PTHrP) negative feedback loop exists that regulates the rate of chondrocyte differentiation [32,33]. Furthermore, multiple groups have demonstrated PTHrP-independent roles for Ihh in promoting chondrocyte hypertrophy and calcification [34,35]. Notably, exogenous Ihh has been shown to induce the expression of type X collagen and MMP-13 in cultured chondrocytes. This observation raises intriguing possibilities, particularly in the context of FOP. However, it remains unclear whether Ihh regulates chondrogenesis in FOP mouse models in a PTHrP-dependent manner. These findings collectively underscore the intricate role of Ihh signaling in the finely tuned processes of chondrogenesis, with implications for both normal skeletal development and pathological conditions such as FOP. Further exploration is needed to elucidate the specific mechanisms involved, especially regarding the potential PTHrP-dependent regulation of chondrogenesis in the context of FOP mouse models.

Further investigation into molecular regulation reveals the involvement of Yap transcriptional activation. Yap is found to be activated in FOP mouse models, and the genetic deletion of *Yap* effectively halts ectopic bone formation, indicating the necessity of it for inducing *Ihh* expression and promoting chondrogenesis. Meanwhile, according to the data from Figure 2, an Ihh mAb treatment could decrease Yap target genes’ expression, as well as Yap activation, suggesting that Yap and Ihh could regulate each other, which is like the Yap-Shh positive feedback loop in heterotopic ossification. Similarly, Yap and Ihh could also form a positive feedback loop to drive chondrogenesis and ectopic bone formation in FOP mouse models. Interestingly, as one of the ligands of Hh signaling, Ihh is stimulated by Yap/Tead4 and Smads coordinating in its promoter. The secreted Ihh could activate Hh singling not only in *Acvr1^R206H^* cells, but also in surrounding wildtype cells, further increasing Yap activation and *Ihh* expression, therefore promoting ectopic bone formation in both cell-autonomous and non-cell-autonomous manners.

Several studies have shown that the function of Yap is often linked to Taz and to downstream genes [36,37,38,39,40]. Vanyai and collaborators demonstrated that the Yap/Taz conditional deletion in chondrocytes from *Col2a1Cre* mice (*Yap^fl/fl^*; *Taz^fl/fl^*; *Col2a1Cre*) resulted in neonatal lethality due, in part, to a cleft palate [41]. They also found chondrodysplasia in Yap/Taz-chondrocyte-specific knockout pups [42]. Moreover, hepatocyte Taz promotes fibrosis by inducing Ihh, a hepatic stellate cell activator, which raises the possibility that Taz could directly regulate Ihh in chondrogenesis [43]. In our study, we cannot exclude the possibility that Yap linked to Taz regulates chondrogenesis and ectopic bone formation in FOP. Based on the Yap deletion data in our FOP mouse models, we found that Yap may play a central role in this process, since the ectopic bone is much reduced after deletion. However, the ectopic bone did not completely disappear after Yap deletion, which indicates that other genes like Taz may also play a role in this process.

Exploring additional methods to treat chondrocyte-related diseases is crucial due to the significant medical challenge posed by abnormal chondrogenesis and the defects observed in conditions like osteoarthritis, which currently have limited treatment options. Recent studies have explored the potential of biotechnological chondroitin (BC) as an alternative to traditional chondroitin sulfate (CS) for treating osteoarthritis [44]. The results indicate that BC, akin to CS, effectively reduces mechanical allodynia, decreases cartilage damage, and attenuates inflammation- and pain-related biochemical markers in an osteoarthritic mouse model induced by MIA (Monosodium Iodoacetate). These findings underscore the promise of BC as a functional ingredient in pharmaceuticals and nutraceuticals for treating cartilage pathologies, suggesting its potential as a viable therapeutic option for addressing osteoarthritis and related conditions [45].

## 5. Conclusions

In conclusion, the identified Yap-Ihh axis emerges as a crucial player in FOP pathogenesis, presenting a promising therapeutic target to prevent and reduce heterotopic ossification in individuals with FOP, thus offering new possibilities for managing this challenging congenital condition through the targeted modulation of Ihh and Yap without affecting normal bone hemostasis.

## 6. Limitations

The study provides valuable insights into the role of the Yap-Ihh axis in driving chondrogenesis and ectopic bone formation in FOP. However, the potential involvement or interaction of Yap with its paralog Taz in this process remains uncertain and warrants further investigation. Another limitation of this study is its exclusive testing of the Yap-Ihh axis in FOP mouse models, which raises questions about the generalizability and applicability of these findings to injury-induced HO mouse models. Additional studies using injury-induced HO mouse models are warranted to validate the relevance of the Yap-Ihh axis as a therapeutic target beyond FOP-specific pathologies.

## Data Availability

Data available on request from the authors.

## Supplementary Materials

The following supporting information can be downloaded at https://www.mdpi.com/article/10.3390/biom14030347/s1, Figure S1: bone mass was not affected by Ihh mAb treatment; Figure S2: bone length was slightly decreased with Ihh mAb treatment; Figure S3: Western blot images of Figure 5D; Table S1: primer sequences for qRT-PCR and ChIP-PCR.

## References

[1] Pignolo R.J., Shore E.M., Kaplan F.S. (2011). Fibrodysplasia ossificans progressiva: Clinical and genetic aspects. Orphanet J. Rare Dis..

[2] Kaplan F.S., Le Merrer M., Glaser D.L., Pignolo R.J., Goldsby R.E., Kitterman J.A., Groppe J., Shore E.M. (2008). Fibrodysplasia ossificans progressiva. Best Pract. Res. Clin. Rheumatol..

[3] Shafritz A.B., Shore E.M., Gannon F.H., Zasloff M.A., Taub R., Muenke M., Kaplan F.S. (1996). Overexpression of an osteogenic morphogen in fibrodysplasia ossificans progressiva. N. Engl. J. Med..

[4] Kaplan F.S., Hahn G.V., Zasloff M.A. (1994). Heterotopic Ossification: Two Rare Forms and What They Can Teach Us. J. Am. Acad. Orthop. Surg..

[5] Kaplan F.S., Shen Q., Lounev V., Seemann P., Groppe J., Katagiri T., Pignolo R.J., Shore E.M. (2008). Skeletal metamorphosis in fibrodysplasia ossificans progressiva (FOP). J. Bone Miner. Metab..

[6] Shore E.M., Xu M., Feldman G.J., Fenstermacher D.A., Cho T.J., Choi I.H., Connor J.M., Delai P., Glaser D.L., LeMerrer M. (2006). A recurrent mutation in the BMP type I receptor ACVR1 causes inherited and sporadic fibrodysplasia ossificans progressiva. Nat. Genet..

[7] Lounev V.Y., Ramachandran R., Wosczyna M.N., Yamamoto M., Maidment A.D., Shore E.M., Glaser D.L., Goldhamer D.J., Kaplan F.S. (2009). Identification of progenitor cells that contribute to heterotopic skeletogenesis. J. Bone Jt. Surg. Am..

[8] Kaplan F.S., Xu M., Seemann P., Connor J.M., Glaser D.L., Carroll L., Delai P., Fastnacht-Urban E., Forman S.J., Gillessen-Kaesbach G. (2009). Classic and atypical fibrodysplasia ossificans progressiva (FOP) phenotypes are caused by mutations in the bone morphogenetic protein (BMP) type I receptor ACVR1. Hum. Mutat..

[9] Shen Q., Little S.C., Xu M., Haupt J., Ast C., Katagiri T., Mundlos S., Seemann P., Kaplan F.S., Mullins M.C. (2009). The fibrodysplasia ossificans progressiva R206H ACVR1 mutation activates BMP-independent chondrogenesis and zebrafish embryo ventralization. J. Clin. Investig..

[10] Culbert A.L., Chakkalakal S.A., Theosmy E.G., Brennan T.A., Kaplan F.S., Shore E.M. (2014). Alk2 regulates early chondrogenic fate in fibrodysplasia ossificans progressiva heterotopic endochondral ossification. Stem Cells.

[11] Hatsell S.J., Idone V., Wolken D.M., Huang L., Kim H.J., Wang L., Wen X., Nannuru K.C., Jimenez J., Xie L. (2015). ACVR1R206H receptor mutation causes fibrodysplasia ossificans progressiva by imparting responsiveness to activin A. Sci. Transl. Med..

[12] Hino K., Ikeya M., Horigome K., Matsumoto Y., Ebise H., Nishio M., Sekiguchi K., Shibata M., Nagata S., Matsuda S. (2015). Neofunction of ACVR1 in fibrodysplasia ossificans progressiva. Proc. Natl. Acad. Sci. USA.

[13] Meng X., Wang H., Hao J. (2022). Recent progress in drug development for fibrodysplasia ossificans progressiva. Mol. Cell. Biochem..

[14] Chakkalakal S.A., Uchibe K., Convente M.R., Zhang D., Economides A.N., Kaplan F.S., Pacifici M., Iwamoto M., Shore E.M. (2016). Palovarotene Inhibits Heterotopic Ossification and Maintains Limb Mobility and Growth in Mice With the Human ACVR1(R206H) Fibrodysplasia Ossificans Progressiva (FOP) Mutation. J. Bone Miner. Res..

[15] Goldring M.B., Tsuchimochi K., Ijiri K. (2006). The control of chondrogenesis. J. Cell. Biochem..

[16] Wada N., Javidan Y., Nelson S., Carney T.J., Kelsh R.N., Schilling T.F. (2005). Hedgehog signaling is required for cranial neural crest morphogenesis and chondrogenesis at the midline in the zebrafish skull. Development.

[17] Shen L., Ma G., Shi Y., Ruan Y., Yang X., Wu X., Xiong Y., Wan C., Yang C., Cai L. (2019). p.E95K mutation in Indian hedgehog causing brachydactyly type A1 impairs IHH/Gli1 downstream transcriptional regulation. BMC Genet..

[18] Zhong L., Huang X., Karperien M., Post J.N. (2015). The Regulatory Role of Signaling Crosstalk in Hypertrophy of MSCs and Human Articular Chondrocytes. Int. J. Mol. Sci..

[19] Luzzi A.J., Ferrer X., Fang F., Golman M., Song L., Marshall B.P., Lee A.J., Kim J.J., Hung C.T., Thomopoulos S. (2023). Hedgehog Activation for Enhanced Rotator Cuff Tendon-to-Bone Healing. Am. J. Sports Med..

[20] St-Jacques B., Hammerschmidt M., McMahon A.P. (1999). Indian hedgehog signaling regulates proliferation and differentiation of chondrocytes and is essential for bone formation. Genes Dev..

[21] Liu F., Massague J., Ruiz i Altaba A. (1998). Carboxy-terminally truncated Gli3 proteins associate with Smads. Nat. Genet..

[22] Retting K.N., Song B., Yoon B.S., Lyons K.M. (2009). BMP canonical Smad signaling through Smad1 and Smad5 is required for endochondral bone formation. Development.

[23] Seki K., Hata A. (2004). Indian hedgehog gene is a target of the bone morphogenetic protein signaling pathway. J. Biol. Chem..

[24] Zanconato F., Forcato M., Battilana G., Azzolin L., Quaranta E., Bodega B., Rosato A., Bicciato S., Cordenonsi M., Piccolo S. (2015). Genome-wide association between YAP/TAZ/TEAD and AP-1 at enhancers drives oncogenic growth. Nat. Cell Biol..

[25] Dey D., Bagarova J., Hatsell S.J., Armstrong K.A., Huang L., Ermann J., Vonner A.J., Shen Y., Mohedas A.H., Lee A. (2016). Two tissue-resident progenitor lineages drive distinct phenotypes of heterotopic ossification. Sci. Transl. Med..

[26] Yang Y., Liu Q., Zhang L., Fu X., Chen J., Hong D. (2021). A modified tape transfer approach for rapidly preparing high-quality cryosections of undecalcified adult rodent bones. J. Orthop. Transl..

[27] Regard J.B., Malhotra D., Gvozdenovic-Jeremic J., Josey M., Chen M., Weinstein L.S., Lu J., Shore E.M., Kaplan F.S., Yang Y. (2013). Activation of Hedgehog signaling by loss of GNAS causes heterotopic ossification. Nat. Med..

[28] Cong Q., Liu Y., Zhou T., Zhou Y., Xu R., Cheng C., Chung H.S., Yan M., Zhou H., Liao Z. (2021). A self-amplifying loop of YAP and SHH drives formation and expansion of heterotopic ossification. Sci. Transl. Med..

[29] McKee C.M., Xu D., Cao Y., Kabraji S., Allen D., Kersemans V., Beech J., Smart S., Hamdy F., Ishkanian A. (2012). Protease nexin 1 inhibits hedgehog signaling in prostate adenocarcinoma. J. Clin. Investig..

[30] Giraud J., Molina-Castro S., Seeneevassen L., Sifre E., Izotte J., Tiffon C., Staedel C., Boeuf H., Fernandez S., Barthelemy P. (2020). Verteporfin targeting YAP1/TAZ-TEAD transcriptional activity inhibits the tumorigenic properties of gastric cancer stem cells. Int. J. Cancer.

[31] Yoon B.S., Pogue R., Ovchinnikov D.A., Yoshii I., Mishina Y., Behringer R.R., Lyons K.M. (2006). BMPs regulate multiple aspects of growth-plate chondrogenesis through opposing actions on FGF pathways. Development.

[32] Lanske B., Karaplis A.C., Lee K., Luz A., Vortkamp A., Pirro A., Karperien M., Defize L.H., Ho C., Mulligan R.C. (1996). PTH/PTHrP receptor in early development and Indian hedgehog-regulated bone growth. Science.

[33] Vortkamp A., Lee K., Lanske B., Segre G.V., Kronenberg H.M., Tabin C.J. (1996). Regulation of rate of cartilage differentiation by Indian hedgehog and PTH-related protein. Science.

[34] Amano K., Ichida F., Sugita A., Hata K., Wada M., Takigawa Y., Nakanishi M., Kogo M., Nishimura R., Yoneda T. (2008). MSX2 stimulates chondrocyte maturation by controlling Ihh expression. J. Biol. Chem..

[35] Mak K.K., Kronenberg H.M., Chuang P.T., Mackem S., Yang Y. (2008). Indian hedgehog signals independently of PTHrP to promote chondrocyte hypertrophy. Development.

[36] Moya I.M., Halder G. (2019). Hippo-YAP/TAZ signalling in organ regeneration and regenerative medicine. Nat. Rev. Mol. Cell Biol..

[37] La Noce M., Stellavato A., Vassallo V., Cammarota M., Laino L., Desiderio V., Del Vecchio V., Nicoletti G.F., Tirino V., Papaccio G. (2021). Hyaluronan-Based Gel Promotes Human Dental Pulp Stem Cells Bone Differentiation by Activating YAP/TAZ Pathway. Cells.

[38] Heng B.C., Zhang X., Aubel D., Bai Y., Li X., Wei Y., Fussenegger M., Deng X. (2020). Role of YAP/TAZ in Cell Lineage Fate Determination and Related Signaling Pathways. Front. Cell Dev. Biol..

[39] Deng Y., Lu J., Li W., Wu A., Zhang X., Tong W., Ho K.K., Qin L., Song H., Mak K.K. (2018). Reciprocal inhibition of YAP/TAZ and NF-κB regulates osteoarthritic cartilage degradation. Nat. Commun..

[40] Zarka M., Hay E., Cohen-Solal M. (2021). YAP/TAZ in Bone and Cartilage Biology. Front. Cell Dev. Biol..

[41] Vanyai H.K., Prin F., Guillermin O., Marzook B., Boeing S., Howson A., Saunders R.E., Snoeks T., Howell M., Mohun T.J. (2020). Control of skeletal morphogenesis by the Hippo-YAP/TAZ pathway. Development.

[42] Li Y., Yang S., Qin L., Yang S. (2021). TAZ is required for chondrogenesis and skeletal development. Cell Discov..

[43] Wang X., Zheng Z., Caviglia J.M., Corey K.E., Herfel T.M., Cai B., Masia R., Chung R.T., Lefkowitch J.H., Schwabe R.F. (2016). Hepatocyte TAZ/WWTR1 Promotes Inflammation and Fibrosis in Nonalcoholic Steatohepatitis. Cell Metab..

[44] Cimini D., Boccella S., Alfano A., Stellavato A., Paino S., Schiraldi C., Guida F., Perrone M., Donniacuo M., Tirino V. (2022). Evaluation of unsulfated biotechnological chondroitin in a knee osteoarthritis mouse model as a potential novel functional ingredient in nutraceuticals and pharmaceuticals. Front. Bioeng. Biotechnol..

[45] Stellavato A., Tirino V., de Novellis F., Della Vecchia A., Cinquegrani F., De Rosa M., Papaccio G., Schiraldi C. (2016). Biotechnological Chondroitin a Novel Glycosamminoglycan With Remarkable Biological Function on Human Primary Chondrocytes. J. Cell. Biochem..

