# Supplementary material for: Hedgehog Signaling Controls Chondrogenesis and Ectopic Bone Formation via the Yap-Ihh Axis
Source: Biomolecules. 2024 Mar 14;14(3):347. doi: 10.3390/biom14030347 (PMC10968511; doi:10.3390/biom14030347)
Supplement: Supplementary file 1 [file biomolecules-14-00347-s001.zip › biomolecules-2879164-supplementary.pdf]

Supplementary Figure 1

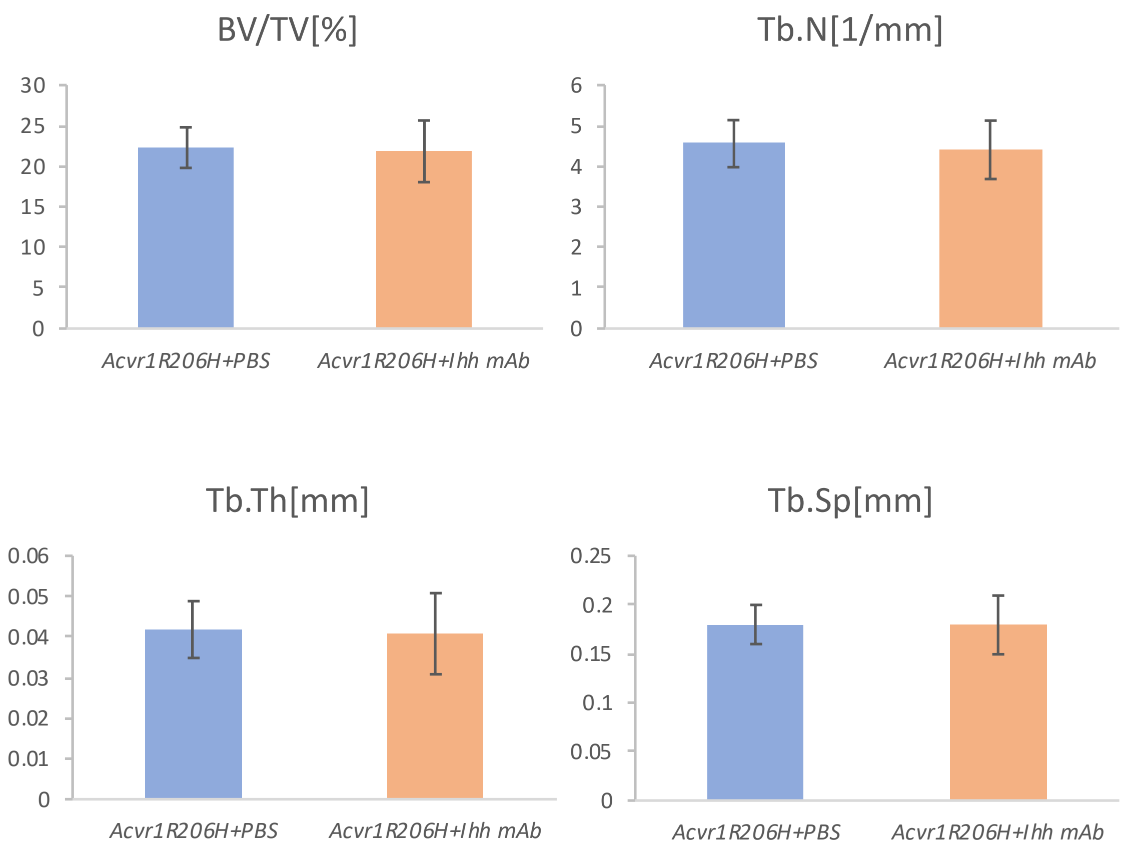

Figure S1. Bone mass is not affected by Ihh mAb treatment. Bone volume fraction (BV/TV), trabecular number (Tb.N), trabecular thickness (Tb.Th), trabecular separation (Tb.Sp) were measured from the indicated mice with PBS or Ihh mAb treatment.

Supplementary Figure 2

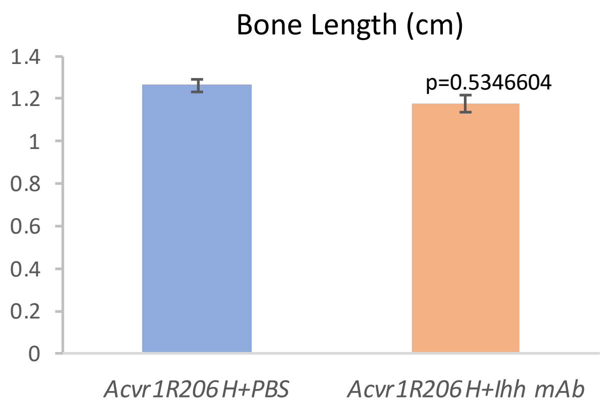

Figure S2. Bone length is slightly decreased with Ihh mAb treatment. Bone length of tibia was measured from indicated mice with PBS or Ihh mAB treatment. P=0.5346604, one-way ANOVA followed by Tukey’s multiple comparisons tests.

Supplementary Figure 3

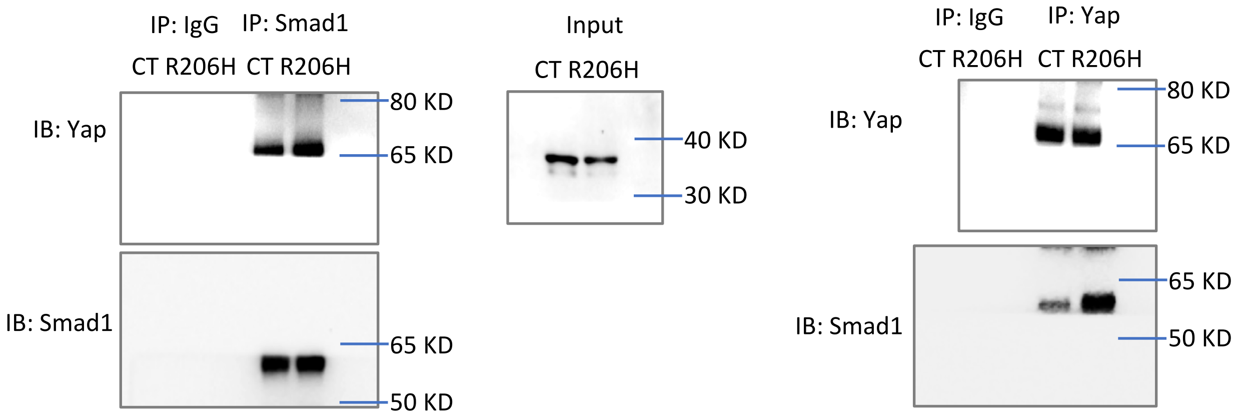

Figure S3. Western Blot images of Figure 5D.

Supplementary Table 1

Table S1: qRT-PCR primer sequences and ChIP-qPCR primer sequences.

qRT-PCR primers	Forward 5’-3’	Reverse 5’-3’
**Gapdh**	GCCTTCCGTGTTCCTACCC	TGCCTGCTTCACCACCTTC
**Hhip**	GGGAAAAACAGGTCATCAGC	ATCCACCAACCAAAGGGC
**Ptch1**	CTCTGGAGCAGATTTCCAAGG	TGCCGCAGTTCTTTTGAATG
**Gli1**	GAAAGTCCTATTCACGCCTTGA	CAACCTTCTTGCTCACACATGTAAG
**Osx**	CCCACTGGCTCCTCGGTTCTCTCC	GCTGAAAGGTCAGCGTATGGCTTC
**Runx2**	AACCCACGGCCCTCCCTGAACTCT	ACTGGCGGGGTGTAGGTAAAGGTG
**Ctgf**	CTGCCTACCGACTGGAAGAC	CATTGGTAACTCGGGTGGAG
**Cyr61**	GCTCAGTCAGAAGGCAGACC	GTTCTTGGGGACACAGAGGA
**Ihh**	ACGTGCATTGCTCTGTCAAGT	CTGGAAAGCTCTCAGCCGGTT
**Shh**	GATGACTCAGAGGTGCAAAGACAA	TGGTTCATCACAGAGATGGCC
**Sox9**	AGTACCCGCATCTGCACAAC	ACGAAGGGTCTCTTCTCGCT
**Col2a1**	ACTGGTAAGTGGGGCAAGAC	CCACACCAAATTCCTGTTCA
**Aggrecan**	GTGGTGGAGCATGCTAGAACCC	ATTCGAGGCTCTTCCCAG

ChIP-qPCR primers	Forward 5’-3’	Reverse 5’-3’
**Primer1**	GGCCGGGTAGCCCGGGA	CCCCAAGAGCCACCCAGA
**Primer2**	ACTCCATGCGGGGGCGCCAT	GCCGGGCAGCTCAGAGTCGA
**Primer3**	ACTCTGAGCTGCCCGGCTCG	CGCGGGTCCCTTCAGTC
**Primer4**	GTTAGCACCCCGGCCCGG	GCGGGTCCCGAGCCCGGAT
**Primer5**	GCAGACCGCACCCTATCCAT	GAGGAGCGCGCGGGACA
**Primer6**	AGCCGCGCGTCCACCCGC	AGATTTGCGCCTGGTG
**Primer7**	CCTGGACAGGAGATGCCA	TGCTTCTCCGCAGGGGACA
**Primer8**	AACCCGAAAGCTGGAGGTCA	GTACTGGGCAGTATAGGGT
**Primer9**	AGAACTAGGGGATGCGGGT	CAGGCTGTTATTCTGTAT
**Primer10**	AAGTGGGGACATCCACAGA	TTAGGTGGACGTCTGCCCG
**Primer11**	ACTGCCTGGGAATCACT	TTCGCTTACTCCCAACA
